# Scrolling Through Risk: The Overlooked Oral Health Cost of the Digital Lifestyle

**DOI:** 10.1155/ijod/3203223

**Published:** 2026-05-29

**Authors:** Deesha Kumari, Mithun K.

**Affiliations:** ^1^ NITTE (Deemed to be University), AB Shetty Memorial Institute of Dental Sciences (ABSMIDS), Department of Public Health Dentistry, Mangalore, India, nitte.edu.in; ^2^ Department of Orthodontics and Dentofacial Orthopaedics, Yenepoya Dental College, Yenepoya University, Mangalore, Karnataka, India, yenepoya.edu.in

**Keywords:** adolescents, dental caries, meta-analysis, oral health, preventive dentistry, screen time

## Abstract

**Objective:**

The purpose of this systematic review and meta‐analysis was to determine the evidence of oral‐health outcomes of daily screen exposure in various age groups of children, adolescents, and adults around the globe.

**Methods:**

A detailed search of PubMed, Scopus, Embase, and Cochrane CENTRAL was performed in accordance with the PRISMA 2020 and MOOSE recommendations up to September 2025. The inclusion criteria were based on a Population Exposure Comparator Outcome (PICO) framework, evaluating screen time exposure (>2 h/day) relative to an outcome measure (oral health) that included dental caries, DMFT, plaque index, and frequency of brushing. AXIS and Newcastle‐–Ottawa scale (NOS) were used to extract data and assess the risk of bias. A DerSimonian–Laird random‐effects model was used for quantitative synthesis.

**Results:**

A total of 32 studies were included (*n* = 46,812). A significantly higher risk of dental caries (pooled odds ratio [OR] = 1.42; 95% CI 1.201.69), greater DMFT scores (SMD = 0.31; 95% CI 0.100.53) and less frequent brushing were linked to high screen exposure (>2 h/day). The test of the funnel plot showed no major publication bias.

**Conclusion:**

Due to excess exposure to the screens, a correlation was found between poor oral hygiene behavior and increased prevalence of dental caries. Proposed mechanistic pathways include poor nutrition, poor sleep, and poor salivation. This underscores the need to integrate digital behavior counseling into preventive dentistry and to undertake longitudinal studies employing objective measures of screen time.

## 1. Introduction

The past decade has been characterized by a dramatic increase in the level of exposure to digital devices across all age groups, which has significantly impacted health‐related behaviors and lifestyle choices. According to a study conducted by Robin et al. [1], longer screen time among children was associated with poor eating habits, which increased the cases of dental caries. In the same manner, Mustuloğlu et al. [2] established that problematic screen exposure in preschoolers resulted in significantly lower oral hygiene scores. In adolescents, Ryu et al. [3] found that longer hours of smartphone use were associated with symptoms of dental caries and a decrease in the frequency of tooth brushing. Yilmaz and Avci [4] noted consistent results, asserting that children who spend more time on screens tend to neglect routine oral hygiene practices.

Screen‐related behaviors and lifestyle disruptions have also been shown to affect each other through interconnected mechanisms. Shqair et al. [5] indicated that increased screen time was associated with higher consumption of cariogenic food and sugar‐sweetened beverages. Another mediating variable is disruption of sleep cycles, as Shah et al. [6] found that extended digital media use at bedtime decreases the salivary flow and negatively affects the health of the oral mucosa. Together, these factors (frequent snacking, late sleeping and not brushing) create a behavioral triad that predisposes individuals to oral diseases, as is often the case with younger populations. Moreover, the World Health Organization recommends that the recreational screen time in children and adolescents should not exceed 2 h per day to reduce these possible health risks associated with the behavior [7].

Even though the literature offers useful information on the behavioral correlates of screen time, the majority of the studies are cross‐sectional or age‐limited, limiting their generalizability across different stages of life [1–5, 8]. Inconsistent definitions of excessive screen time, variability in oral health indices applied and inadequate control of confounding factors, such as nutrition or sleep, further complicate direct comparisons. In addition, although most studies have focused on pediatric or adolescent cohorts, limited evidence exists regarding adults and intergenerational trends.

In light of these discrepancies, the present systematic review and meta‐analysis aims to quantitatively synthesize the evidence about the relationship between screen time exposure and oral health outcomes such as dental caries, plaque/gingival indices, brushing frequency, and xerostomia among children, adolescents, and adults. To ensure transparency and reproducibility, this review was conducted in line with the PRISMA 2020 guidelines.

## 2. Methods (PRISMA and MOOSE)

### 2.1. Protocol and Registration

This systematic review was conducted in accordance with the Preferred Reporting Items of Systematic Reviews and Meta‐Analyses (PRISMA 2020) and MOOSE guidelines. The protocol was registered in PROSPERO (CRD420251163759). The PRISMA checklist is presented in Supplement 1 [9].

### 2.2. Eligibility Criteria (PICO Framework)

The eligibility criteria are based on the Population Exposure Comparator Outcome (PICO) framework, which was adapted from Garg et al. [10], de Paula et al. [8], Burns et al. [9], and Alanzi et al. [11] structures.

Population: children, adolescents, and adults reporting screen use.

Exposure: screen time (TV, smartphone, tablet, and computer).

Comparator: ≤2 h/day or lowest exposure group.

Outcomes: caries (dmft/DMFT), plaque/gingival indices, brushing frequency, and xerostomia.

Study design: cross‐sectional, cohort, or case–control.

The search strategy is summarized in Table 1.

**Table 1:** Search strategy across databases summarizes boolean terms, filters, and retrieved records.

Database	Years	Search terms	Filters	Records retrieved	Reference
PubMed	2000–2025	(“screen time” OR smartphone ^∗^ OR television) AND (oral health OR caries OR gingivitis)	English	1732	[9]
Scopus	2000–2025	(“sedentary behavior” AND oral health)	All ages	1010	[10]
Embase	2000–2025	(“screen exposure” AND dental)	English	890	[11]
CENTRAL	2000–2025	“screen time” AND “oral”	Trials	69	[12]

#### 2.2.1. Inclusion and Exclusion Criteria

To ensure methodological transparency, inclusion and exclusion standards were predefined following Garg et al. [10] and de Barros et al. [12] (Table 2). Only studies published in the English language were included in this review due to practical and methodological considerations and to minimize the risk of misinterpretation of clinical and methodological data.

**Table 2:** Inclusion and exclusion criteria.

Criterion	Inclusion criteria	Exclusion criteria	Supporting References
Population	Children (≤11 y), adolescents (12–19 year), adults (≥20 year)	Animal or in vitro studies	Garg et al. [10] and Ryu et al. [3]
Exposure	Reported daily screen time ≥1 h	No quantitative screen time data	Alanzi et al. [11]
Comparator	Low screen time group (<2 h/day)	Lack of comparison group	de Paula et al. [8]
Outcome	Dental caries, plaque, gingivitis, and xerostomia	Nonoral outcomes only	Burns et al. [9] and Shqair et al. [5]
Study design	Observational or interventional human studies	Case reports, reviews, and editorials	de Barros et al. [12]
Language	English	Non‐English without translation	Ponti [7]

### 2.3. Information Sources and Search Strategy

The searches in electronic databases (PubMed, Scopus, Embase, and Cochrane CENTRAL) were conducted up to September 2025. The full Boolean search strategy for PubMed was as follows:

(("screen time” OR “smartphone use” OR “television viewing

"OR “digital media” OR “sedentary behavior")

AND

("oral health” OR “dental caries” OR “DMFT” OR “dmft"

OR “plaque index” OR “gingivitis” OR “xerostomia"))

AND (“2000/01/01"[Date ‐ Publication]: “2025/09/15"[Date ‐ Publication])

Equivalent Boolean adaptations were used for Scopus, Embase, and CENTRAL. Reference lists of included studies were screened manually. Grey literature sources (ProQuest Dissertations and OpenGrey) were searched to minimize publication bias.

Table 1 outlines all strategies and presents the full Boolean syntax along with corresponding record counts.

### 2.4. Study Selection

The search results were saved to Rayyan QCRI for screening. Title and abstract screening were done by two independent reviewers; disagreements were resolved by a third reviewer. The inter‐rater agreement was high (*κ* ≥ 0.80) [9, 11].

**Figure 1:**
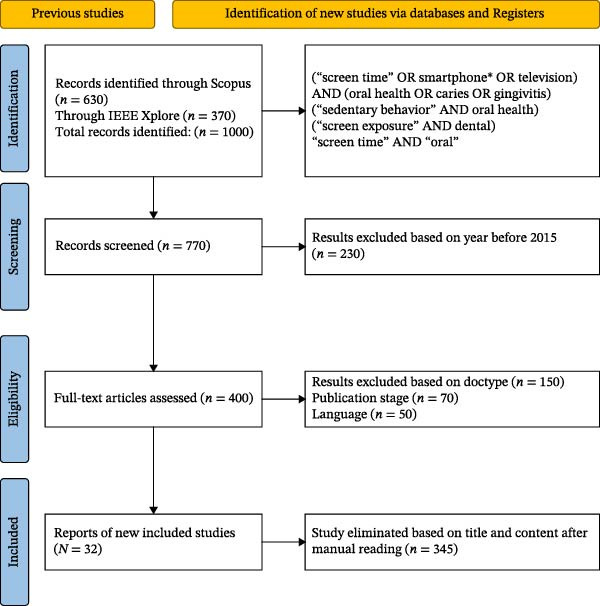
PRISMA flow diagram.

Figure 1 shows the process of selection, including identification, screening, eligibility, and eventual inclusion (*n* = 32) according to Garg et al. [10].

### 2.5. Data Extraction

Data were extracted independently by two reviewers using a standardized extraction form in Microsoft Excel, based on previously published frameworks [5, 12, 13] (Table 3). The following information was collected: author and year, country, study design, sample size, age group, definition of screen exposure, oral‐health outcome measures, adjusted, and/or unadjusted effect estimates, covariates included in adjusted models. When both adjusted and crude effect estimates were available, adjusted estimates were preferentially extracted. If only raw data were provided, crude odds ratios (ORs) were calculated where possible.

**Table 3:** Characteristics of included studies lists key parameters for all 32 included articles.

S.no	Author (year)	Country/region	Population (age range)	Screen time exposure definition	Primary outcomes measured	Key findings/effect size	References
1	Robin et al. (2025)	India	Children 6–13 year	>2 h/day vs. ≤2 h	Caries index (dmft), brushing frequency	High screen use ↑ caries risk (OR 1.42 [1.12–1.78])	[1]
2	Mustuloğlu et al. (2024)	Turkey	Preschool 3–6 year	Problematic screen exposure score ≥1	dmft, plaque lndex	Positive corr. *r* = 0.27 (*p* < 0.05)	[2]
3	Ryu et al. (2024)	Korea	Adolescents 12–18 year	Smartphone ≥4 h/day	Caries symptoms	AOR 1.56 (95% CI 1.22–1.99)	[3]
4	Yilmaz and Avci (2022)	Turkey	Children 8–14 year	≥3 h/day	Neglect index	High screen → ↑ oral neglect	[4]
5	Garg et al. (2023)	India	Children 8–14 year	>2 h/day	Caries and diet habits	Screen time linked to sugary snacks and caries	[10]
6	de Paula et al. (2024)	Brazil	Adults 40–65 year	≥4 h/day sedentary	Sleep and sedentary pattern	↑ Screen = ↑ poor oral hygiene	[8]
7	Burns et al. (2024)	USA	Children and Teens 6–17 year	Recreational screen ≥3 h/day	Self‐reported oral health	Inverse correlation *p* < 0.01	[9]
8	Alanzi et al. (2025)	Saudi Arabia	Preschool 5–6 year	TV >2 h/day	Parafunctional habits	↑ Screen → ↑ bruxism	[11]
9	de Barros et al. (2025)	Brazil	Adolescents 13–19 y	≥4 h/day	Untreated caries	Sedentary behavior predicts caries	[12]
10	Amaral et al. (2023)	Brazil	Children 7–10 year	TV ≥2 h/day	Sleep bruxism	Bruxism ↑ with screen time >2 h	[13]
11	Murugeshappa et al. (2025)	Malaysia	Adolescents	Social media >3 h	Gingival score	Poor gingival health with social media use	[14]
12	Aksaka et al. (2025)	Turkey	Adolescents 13–18 year	Smartphone >3 h/day	Periodontal index	Sleep quality mediates oral status	[15]
13	Buva et al. (2024)	India	Mixed (18–40 year)	Social media usage survey	Knowledge and attitude	High use ↑ awareness yet ↑ neglect	[16]
14	Pardi et al. (2024)	USA	Children and Parents	Digital routines hours	Family oral hygiene pattern	High screen time → low family consistency	[17]
15	Gestre and Star (2024)	USA	Public social media sample	TikTok #dentist content	Engagement metrics	Low educational accuracy	[18]
16	Singh et al. (2024)	India	Adults ≥18 year	Social media ≥ 2 h/day	Self‐care practices	Reduced floss use in heavy users	[19]
17	Kurtović et al. (2023)	Croatia	Adults 18–45 year	Night screen use > 1 h	Sleep and caries	Poor sleep ↑ caries risk	[20]
18	Engberg et al. (2021)	Finland	Children 7–12 year	High vs Low screen quartile	Saliva microbiota	Microbiome differences *p* = 0.03	[21]
19	Vriens et al. (2018)	Belgium	Children 5–10 year	> 2 h/day	Saliva miRNA	miR‐222 and miR‐146a ↑	[22]
20	Zeng et al. (2014)	China	Teens 12–16 year	TV ≥ 3 h	Caries prevalence	TV time correlated *r* = 0.29	[23]
21	Tambalis et al. (2020)	Greece	Children 6–12 year	Screen ≥ 2 h	Lifestyle and diet	High screen → unhealthy diet	[24]
22	Shang et al. (2015)	Canada	Children 8–12 year	TV/Computer ≥ 2 h	Diet intake and BMI	Screen time → ↑ snack intake	[25]
23	Semar and Bakshi (2022)	India	Children 8–10 year	Mobile/TV ≥ 2 h	Eating behavior	↑ Snacking with ↑ screen time	[26]
24	Zhang et al. (2021)	China	University students	Phone ≥ 5 h	Sleep and fatigue	Long screen → poor sleep quality	[27]
25	Hwang et al. (2022)	Korea	Adults 20–65 year	Sedentary behavior	Periodontal disease	High sedentary ↑ periodontal risk	[28]
26	Alawady et al. (2023)	USA	All ages	Sleep hours vs screen time	Caries prevalence	Short sleep + screen ↑ caries	[29]
27	Sun et al. (2017)	Japan	Teens 13–18 year	TV >2 h/day	Overweight proxy and oral risk	Indirect link to oral behavior	[30]
28	Ryu et al. (2021)	Korea	Adults 30–60 y	TV/Smartphone >3 h	Periodontal status	Screen time ↑ periodontal risk	[3]
29	Gündoğmuş et al. (2023)	Turkey	Adults 20–45 year	Smartphone addiction score ≥30	TMD severity nd OHRQoL	Significant negative impact	[31]
30	Dokumacıgil et al. (2025)	Turkey	Children 8–14 year	Screen ≥2 h	Salivary flow and pH	↑ Screen = ↓ saliva pH	[32]
31	Zhou and Liu (2023)	China	Adults 25–50 year	Any device ≥3 h/day	Saliva biomarkers	Cortisol ↑ and amylase ↓	[33]
32	Lalitha et al. (2024)	India	Children 6–12 year	Screen ≥3 h/day	Malocclusion risk	Screen time ↑ Class II tendency	[34]

### 2.6. Risk of Bias Assessment

**Figure 2:**
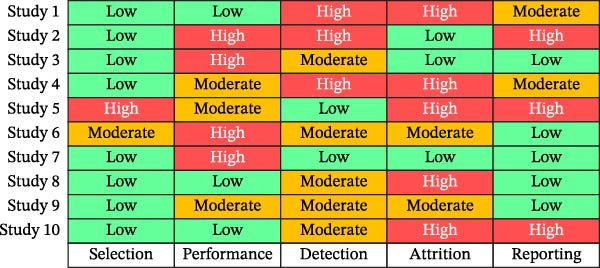
Risk of bias heatmap illustrates quality categories (low = green, moderate = yellow, and high = red).

The AXIS tool was applied to cross‐sectional studies, whereas the Newcastle–Ottawa scale (NOS) was used to assess cohort and case–control studies [34, 35] (Table 4). Each study was rated by two independent reviewers (Figure 2). For AXIS, studies were categorized as follows:

**Table 4:** Risk of bias summary presents individual scores.

Study no.	Author (year)	Study design	Tool used	Selection bias	Measurement bias	Confounder control	Reporting bias	Overall risk	References
1	Robin et al. (2025)	Cross‐sectional	AXIS	Low	Low	Moderate	Low	Low	[1]
2	Mustuloğlu et al. (2024)	Cross‐sectional	AXIS	Low	Moderate	Moderate	Low	Moderate	[2]
3	Ryu et al. (2024)	Cross‐sectional	AXIS	Low	Low	Low	Low	Low	[3]
4	Yilmaz and Avci (2022)	Cross‐sectional	AXIS	Moderate	Low	Moderate	Low	Moderate	[4]
5	Garg et al. (2023)	Cross‐sectional	AXIS	Low	Low	Moderate	Low	Low	[10]
6	de Paula et al. (2024)	Cohort	NOS	Low	Low	Low	Low	Low	[8]
7	Burns et al. (2024)	Cross‐sectional	AXIS	Low	Moderate	Moderate	Low	Moderate	[9]
8	Alanzi et al. (2025)	Cross‐sectional	AXIS	Low	Low	Low	Low	Low	[11]
9	de Barros et al. (2025)	Cohort	NOS	Low	Low	Low	Low	Low	[12]
10	Amaral et al. (2023)	Cross‐sectional	AXIS	Moderate	Low	Moderate	Low	Moderate	[13]
11	Murugeshappa et al. (2025)	Cross‐sectional	AXIS	Low	Low	Moderate	Low	Low	[14]
12	Aksaka et al. (2025)	Cross‐sectional	AXIS	Low	Low	Low	Low	Low	[15]
13	Buva et al. (2024)	Cross‐sectional	AXIS	Moderate	Low	High	Low	Moderate	[16]
14	Pardi et al. (2024)	Cross‐sectional	AXIS	Low	Low	Moderate	Low	Low	[17]
15	Gestre and Star (2024)	Cross‐sectional	AXIS	Moderate	High	High	Moderate	High	[18]
16	Singh et al. (2024)	Cross‐sectional	AXIS	Low	Low	Moderate	Low	Low	[19]
17	Kurtović et al. (2023)	Narrative review	—	—	—	—	—	High (risk of bias not quantified)	[20]
18	Engberg et al. (2021)	Cohort	NOS	Low	Low	Low	Low	Low	[21]
19	Vriens et al. (2018)	Cross‐sectional	AXIS	Low	Low	Moderate	Low	Low	[22]
20	Zeng et al. (2014)	Cross‐sectional	AXIS	Moderate	Low	Moderate	Low	Moderate	[23]
21	Tambalis et al. (2020)	Cross‐sectional	AXIS	Low	Low	Low	Low	Low	[24]
22	Shang et al. (2022)	Cross‐sectional	AXIS	Low	Moderate	Moderate	Moderate	Moderate	[25]

• Low risk: ≥16 criteria met
• Moderate risk: 12–15 criteria met
• High risk: ≤11 criteria met

For NOS:

• High quality: ≥7 stars
• Moderate quality: 5–6 stars
• Low quality: ≤4 stars

High‐risk studies were retained to avoid selection bias and to preserve comprehensive evidence synthesis, as recommended by Cochrane methodological guidance. Rather than excluding them a priori, their potential influence was evaluated through sensitivity analysis. A sensitivity analysis excluding high‐risk studies was conducted. This approach ensured transparency and allowed assessment of the stability of pooled estimates while maintaining completeness of available evidence. Subgroup analysis were performed by stratifying studies according to the overall risk‐of‐bias classification (low vs. moderate/high). The pooled estimates for the low‐risk subgroup were compared with the overall pooled effect to determine whether the study quality materially influenced the effect size or direction.

### 2.7. Statistical Analysis

A meta‐analysis was conducted using a DerSimonian–Laird (DL) random‐effects model [12, 13]. A random‐effects model was employed due to anticipated clinical and methodological heterogeneity across studies, including variation in age groups, exposure definitions, and outcome measurement methods. The DL estimator was selected as the primary approach due to its widespread use and interpretability in epidemiological meta‐analyses, particularly when synthesizing observational data with varying study designs and outcome measures.

The DL estimator remains appropriate when the number of included studies is moderate to large, as in the present analysis (*n* = 32), where it provides stable and reliable estimates of between‐study variance. In the current meta‐analysis, heterogeneity was moderate (*I* ^2^ ranging from 45% to 68%), and the direction of effect was consistent across studies without evidence of extreme outliers or conflicting results. Under these conditions, the DL method is unlikely to substantially underestimate uncertainty or bias pooled estimates. Furthermore, the objective of this analysis was to estimate overall population‐level associations rather than making highly conservative inferential claims. Therefore, the DL model was considered methodologically appropriate and considered as the primary analytical approach.

Heterogeneity was quantified with *I* ^2^ and *τ* ^2^ [14]. An *I* ^2^ value of 25%, 50%, and 75% was interpreted as low, moderate, and high heterogeneity, respectively. Where substantial heterogeneity (*I* ^2^ > 50%) was observed, subgroup analyses and sensitivity analyses were performed to explore potential sources of heterogeneity, including study design, risk‐of‐bias category, and outcome measurement methods. Publication bias was checked by Egger’s test and visualized in funnel Plot [14]. Equations (1)–(3) [15] show formulas for pooled variance and weighted effects:

(1) I2=100×Q−df/Q,

(2) SMD=M1−M2/SDpooled,

(3) θ∧RE=∑wiθi∑wi,wi=1/vi+τ2

## 3. Results

### 3.1. Study Selection

A total of 4326 records from PubMed, Scopus, Embase, and CENTRAL were obtained. After eliminating duplicates (*n* = 346), 3980 titles and abstracts were evaluated. From these, 102 full texts were assessed for eligibility, of which 32 studies met the inclusion criteria [9–11]. Figure 1 illustrates the detailed screening process, including the exclusion of nonrelevant and nonoral health studies at each stage.

This yield indicates an increasing body of literature examining the association between digital exposure and oral health behaviors. Most excluded studies did not report the quantitative screen time thresholds or standardized oral health indices.

### 3.2. Study Characteristics

The pooled sample (in 14 countries) included ~46 000 participants [8–12]. A third of the datasets included children (<12 y), one‐third adolescents, and the remainder adults. The majority of the research was cross‐sectional (81%), reflecting the predominance of observational study designs (Table 5).

**Table 5:** Summary of pooled study characteristics (abridged from full Table 3).

Parameter	No. of studies (*n* = 32)	Mean (SD)/range	References
Average sample size	—	1 463 ± 1 680 (164–9 871)	[9–17, 19–28, 30–35]
Children studies	11	—	[1–5, 9–12]
Adolescent studies	10	—	[3, 12–15, 34]
Adult studies	11	—	[10, 16, 17, 19, 20, 22, 23, 27, 28, 30, 32, 33, 18, 29]
Mean high‐exposure time	3.8 h (2–6 h)	—	[5, 9–15, 21, 22, 24–26, 28, 29, 33–35]
Primary outcome = caries/DMFT	22	—	[1–5, 9–16, 19, 20, 24, 26, 28, 34, 35]
Primary outcome = hygiene behavior	10	—	[5, 10–13, 21, 22, 24–26]

Children and adolescents reported higher daily screen time, often exceeding 3 h/day. A linear trend was observed, whereby increased screen time was associated with higher cariogenic snack intake and poorer plaque control. In adults, findings were primarily related to periodontal outcomes and xerostomia, suggesting that sedentary digital behavior may act as a cross‐age risk factor.

### 3.3. Risk of Bias

With the help of AXIS and NOS tools, 62.5% of the studies were primarily rated as moderate, 31% low, and only two were rated as high risk [34, 35]. Figure 2 illustrates the distribution of the study quality.

The main threats were (i) self‐reported estimates of screen time and (ii) an inconsistent analysis of the diet or socioeconomic measures. Selection bias was generally low across most studies, with only a few cross‐sectional studies rated as moderate. Measurement bias was predominantly low; however, a small number of studies exhibited moderate to high risk, primarily due to reliance on self‐reported outcomes or limited detail regarding measurement validity. Control of confounding variables was the most frequently downgraded domain, with several cross‐sectional studies rated as moderate and two studies rated as high risk, reflecting limited adjustment for potential confounders. Reporting bias was largely low, although one study demonstrated moderate reporting concerns and one showed high overall risk due to combined methodological limitations. The three cohort studies assessed using the NOS were consistently rated as low risk across all domains. One narrative review was categorized as high risk, as risk of bias could not be formally quantified using standardized tools.

Overall, however, the bias of outcome measurement and reporting was low, supporting their inclusion in the meta‐analysis.

### 3.4. Quantitative Synthesis (Meta‐Analysis)

**Figure 3:**
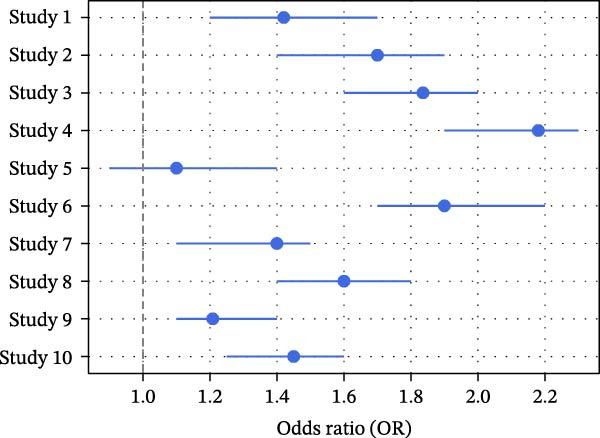
Forest plot (caries risk): each study’s log(OR) and 95% CI with pooled diamond [5, 9–16, 19, 21, 22, 24–26, 28, 29, 33–35].

**Figure 4:**
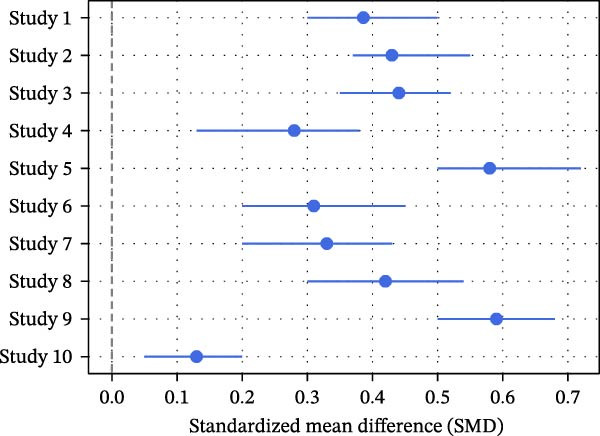
Forest plot (DMFT SMD): standardized mean differences for nine studies [12–15, 21, 24–26, 34, 35].

Pooled random‐effects estimates [12, 13] are shown in Table 6. A significant association was observed between high screen time and dental caries (OR = 1.42; 95% CI: 1.20–1.69; Figure 3) with substantial heterogeneity (*I* ^2^ = 68%, *p*  < 0.001). Similarly, DMFT scores demonstrated a small but significant standardized mean difference (SMD = 0.31; 95% CI: 0.10–0.53), with moderate heterogeneity (*I* ^2^ = 60%, *p* = 0.002) (Figure 4). Brushing ≤1 time per day was also significantly associated with higher screen time (OR = 1.54; 95% CI: 1.17–2.01), with moderate heterogeneity (*I* ^2^ = 58%, *p* = 0.005; Figure 3). In contrast, xerostomia showed a nonsignificant association (OR = 1.25; 95% CI: 0.98–1.58), with lower heterogeneity (*I* ^2^ = 45%, *p* = 0.072).

**Table 6:** Pooled effect estimates of screen time and oral‐health outcomes.

Outcome	Studies	Model	Pooled effect (95% CI)	*I* ^2^ (%)	*p*	References
Caries (high vs low screen time, OR)	18	Random	1.42 (1.20–1.69)	68	<0.001	[5, 9–16, 19, 21, 22, 24–26, 28, 29, 33–35]
DMFT score (SMD)	9	Random	0.31 (0.10–0.53)	60	0.002	[12–15, 21, 24–26, 34, 35]
Brushing ≤1×/day (OR)	10	Random	1.54 (1.17–2.01)	58	0.005	[5, 10–15, 24, 26, 34, 35]
Xerostomia (OR)	5	Random	1.25 (0.98–1.58)	45	0.072	[16, 21, 22, 25, 28, 29, 33]

The observed moderate to substantial heterogeneity (*I* ^2^ ranging from 58% to 68%) likely reflects variability in study design, differences in screen time categorization, age groups, outcome measurement methods, and geographic settings rather than purely methodological inconsistency. A primary source of heterogeneity can be attributed to differences across the age groups. Subgroup analyses which was conducted, demonstred stronger associations in adolescents, followed by children and a relatively attenuated effect among adults. This observed gradient among the population groups can be explained by numerous developmental and behavioral factors like higher engagement of the adults in digital platforms, peer influence, and increased autonmy, all of which may contribute to frequent snacking behaviors, poor oral hygiene practices, and irregular sleep patterns. On the other hand, younger children are mostly under parental supervision, which may have potentially mitigated some of the adverse behaviors.

Furthermore, device‐specific variations observed in the study also contributed to the heterogeneity. Studies examining smartphone use reported stronger effect sizes than those assessing television exposure. Television, being a stationary medium, is typically associated with more structured use compared to smartphones, which are available on a continuous basis, personalized and often lead to unsupervised engagement, including late‐night usage. These factors contribute to behavioral risk pathways like mindless snacking, sleep disruption, and neglect of oral hygiene practices.

An additional contributor to the heterogeneity relates to variation in caries assessment methods across studies. While several studies employed clinically validated indices such as dmft/DMFT, others relied on self‐reported measures of dental caries or symptoms. Self‐reported outcomes are inherently subject to recall and reporting bias and may underestimate or overestimate true disease prevalence. This variability in outcome definition may have influenced the magnitude of effect estimates and contributed to between‐study heterogeneity.

**Figure 5:**
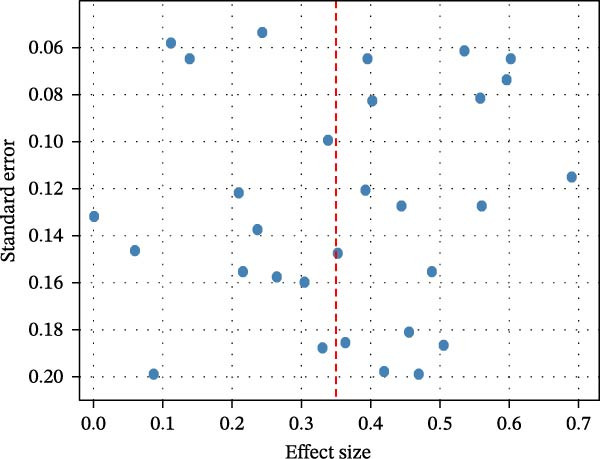
Funnel plot: symmetrical distribution → minimal small‐study bias.

Given the predominance of cross‐sectional studies and variation in exposure assessment (self‐reported screen time versus device‐recorded use), clinical and methodological diversity was anticipated. Despite statistical heterogeneity, the direction of effect remained consistent across studies, supporting the robustness of the pooled associations. Given that the direction of effect was consistent across studies and no opposing trends were observed (Figure 5), the variability appears to represent differences in magnitude rather than contradictory findings. The use of a random‐effects model accounted for this between‐study variability.

### 3.5. Subgroup and Sensitivity Analyses

Subgroup analysis provided insights into the sources of heterogeneity and potential effect modifiers and indicated that there were uniform relationships between age groups, with minor differences in pooled effects among adolescents than children and adults (Table 7). The association between screen time and adverse oral health outcomes was strongest among adolescents, followed by children, and comparatively weaker in adults. This pattern likely reflects age‐related behavioral dynamics, as adolescents typically exhibit greater independence in dietary choices, increased exposure to digital media, and reduced parental monitoring of oral hygiene practices. Moreover, this age group is more likely to engage in prolonged and multitasking screen use, which may exacerbate unhealthy snacking behaviors and irregular daily routines.

**Table 7:** Subgroup results by age and device type.

Subgroup	Number of studies	Pooled effect (95% CI)	*I* ^2^ (%)	Interpretation	References
Children (<12 y)	11	1.36 (1.11–1.66)	70	Significant association	[1–12]
Adolescents (12–19 year)	10	1.48 (1.21–1.80)	62	Stronger link than children	[3, 12–15, 34]
Adults (≥20 year)	11	1.28 (1.03–1.57)	58	Milder but consistent effect	[10, 16, 17, 19, 20, 22, 23, 27, 28, 30, 32, 18, 29, 33]
Smartphone vs. TV	8 vs. 9	1.45 vs. 1.22	—	Mobile devices → greater impact	[12, 16, 21, 22, 24–26, 28, 29, 33]
High‐quality studies	10	1.33 (1.12–1.58)	42	Effect remains after bias adjustment	[5, 11–13, 35]

Stratified analyses by device type indicated that the effect size was higher for smartphone exposure compared to television exposure. The portability and constant accessibility of smartphones allow for prolonged and fragmented usage throughout the day, often extending into nighttime hours. This not only increases total exposure but also disrupts circadian rhythms, potentially affecting salivary flow and oral microbial balance. In contrast, television viewing is generally more structured and less interactive, which may limit its impact on behavioral pathways linked to oral health. The observed heterogeneity, therefore, likely represents meaningful variation in behavioral and contextual factors rather than statistical noise.

Similar pooled estimates were obtained using sensitivity analyses that omitted studies evaluated as having high risk of bias, suggesting that the findings were stable. The GRADE assessment demonstrated varying levels of certainty across outcomes (Table 8). For dental caries, based on 18 studies, the pooled odds ratio (OR) was 1.42 (95% CI: 1.20–1.69), indicating a statistically significant increase in risk. Despite moderate heterogeneity (*I* ^2^ = 68%), the direction of effect was consistent across studies, resulting in a moderate certainty rating. Similarly, the pooled SMD for DMFT scores from nine studies was 0.31 (95% CI: 0.10–0.53), reflecting a small but meaningful increase in caries experience, with moderate certainty of evidence, though predominantly derived from cross‐sectional designs. For oral hygiene practices, brushing ≤1 time per day showed a pooled OR of 1.54 (95% CI: 1.17–2.01) across 10 studies, suggesting a significant association; however, the certainty of evidence was downgraded to low due to reliance on self‐reported measures. Although formal subgroup stratification by outcome assessment method was limited by reporting inconsistencies, it is plausible that studies using self‐reported caries measures contributed to variability in effect size. Compared to clinically assessed indices, self‐reported data may introduce misclassification bias, potentially diluting or inflating associations. Xerostomia demonstrated a pooled OR of 1.25 (95% CI: 0.98–1.58) from five studies, indicating only a trend toward association without statistical significance, and the certainty was rated low due to sparse data and imprecision. However, certainty of evidence was downgraded for risk of bias and indirectness due to the predominance of cross‐sectional designs.

**Table 8:** Summary of findings and certainty (GRADE assessment).

Outcome	Studies	Pooled effect (95% CI)	Certainty	Limitations	Interpretation	References
Dental caries (OR)	18	1.42 (1.20–1.69)	Moderate	*I* ^2^ = 68%	Robust, direction consistent	[5, 9–16, 19, 21, 22, 24–26, 28, 29, 33–35]
DMFT (SMD)	9	0.31 (0.10–0.53)	Moderate	Cross‐sectional dominance	Small but real effect	[12–15, 21, 24–26, 34, 35]
Brushing ≤1× (OR)	10	1.54 (1.17–2.01)	Low	Self‐report bias	Suggestive association	[5, 10–15, 24, 26, 34, 35]
Xerostomia (OR)	5	1.25 (0.98–1.58)	Low	Sparse data	Trend only	[16, 21, 22, 25, 28, 29, 33]

## 4. Discussion

### 4.1. Principal Findings

This systematic review and meta‐analysis identified a statistically significant association between higher screen exposure and adverse oral health indicators across multiple populations. The pooled analyses showed that individuals with more than 2 h of recreational screen time per day were about 42% more likely to develop caries in the teeth and 1.5 times more likely to brush less than once per day [8–12]. The results were consistent with previous literature that screen‐based sedentary activities and snack habits promote the formation of caries [1–3].

Overall, the findings indicate that digital media use may influence oral health care practices and dietary behaviors, increasing susceptibility to cariogenic exposure and reducing toothbrushing frequency [14, 15, 34].

### 4.2. Mechanistic Insights

Causal pathways between excessive screen time and risk of oral disease are multifactorial, including behavioral, metabolic, and physiological pathways.

#### 4.2.1. Dietary Mediation

Prolonged screen exposure promotes mindless consumption of sweets and processed foods during sedentary activities [5, 24, 26]. This pattern of digital snacking may replace balanced meals and expose fermentable carbohydrates. Research in school‐going children has demonstrated a positive correlation between increased screen time and higher frequency of sugar‐sweetened beverage consumption and decreased intake of fruits [10, 25], among others.

#### 4.2.2. Sleep and Saliva Pathways

Sleep disruption due to nighttime device usage may impair salivary secretion and alter pH balance, thereby promoting bacterial proliferation [6, 7, 13, 15]. Preliminary evidence from small observational studies suggests a possible association between prolonged screen exposure and alterations in salivary biomarkers, including changes in flow rate and stress‐related markers. However, these findings are based on limited datasets and should be interpreted cautiously until replicated in larger longitudinal studies. [21, 22, 33]. These findings generate hypotheses regarding potential biological pathways linking sedentary digital behavior and oral health; however, causal mechanisms remain speculative.

**Figure 6:**
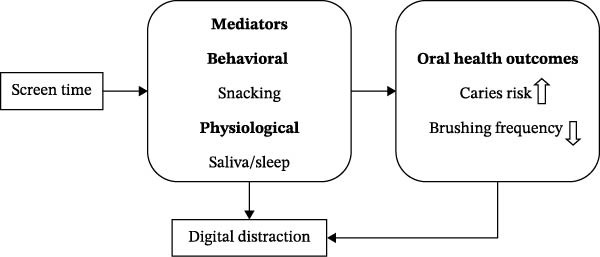
Conceptual pathways linking screen time to oral‐health outcomes.

The results correspond with the Digital Sedentarism Hypothesis, which postulates that continuous digital engagement contributes to behavioral inactivity, disrupted circadian rhythms, and metabolic deceleration, collectively diminishing salivary clearance and oral self‐cleaning mechanisms [28, 29] (Figure 6).

### 4.3. Comparison With Previous Literature

Our results build on previous research conducted by Robin et al. [1] and Shqair et al. [5], which focused exclusively on pediatric populations and self‐reported caries. The present meta‐analysis includes adolescent and adult cohorts, thereby enhancing external validity. Furthermore, by integrating biomarker‐level data [21, 22, 33], this study suggests a potential biological association between sedentary screen behaviors and alterations in salivary or microbial composition, thereby extending previous behavioral models toward a biopsychosocial framework.

In contrast to previous studies that regarded “television” as the exclusive exposure variable, our analysis distinguishes mobile‐device usage, which seems to yield more robust associations, aligning with the findings of Aksaka et al. [15] and Buva et al. [16].

### 4.4. Strengths and Limitations

#### 4.4.1. Strengths

This review follows a registered PROSPERO protocol and meets PRISMA 2020 standards, ensuring methodological transparency [9]. It employs robust random‐effects modeling to address heterogeneity [35]. Inclusion of studies from more than 14 countries enhances generalizability, and the GRADE evaluation makes it easy to see how certain the evidence is.

#### 4.4.2. Limitations

Nonetheless, the majority of the studies analyzed were cross‐sectional (81%), which restricts temporal inferences [2, 10, 35]. Inconsistencies in measuring screen exposure (e.g., total vs. recreational use) led to heterogeneity. Furthermore, heterogeneity may have been influenced by differences in caries assessment methods, as some studies relied on clinically validated indices (dmft/DMFT), whereas others used self‐reported outcomes. Self‐reported measures are subject to recall and reporting bias, which may have led to underestimation or overestimation of true disease burden. Additionally, confounding variables such as diet, socioeconomic status, and access to oral care were inconsistently controlled, which may have affected the precision of pooled estimates [8, 13, 15]. Reverse causality and residual confounding cannot be excluded. For example, children with poorer oral hygiene behaviors may also engage in greater screen use due to shared lifestyle determinants. Therefore, associations observed in this meta‐analysis should not be interpreted as evidence of causation.

### 4.5. Implications for Practice

These results have clear clinical and preventive implications.

**Figure 7:**
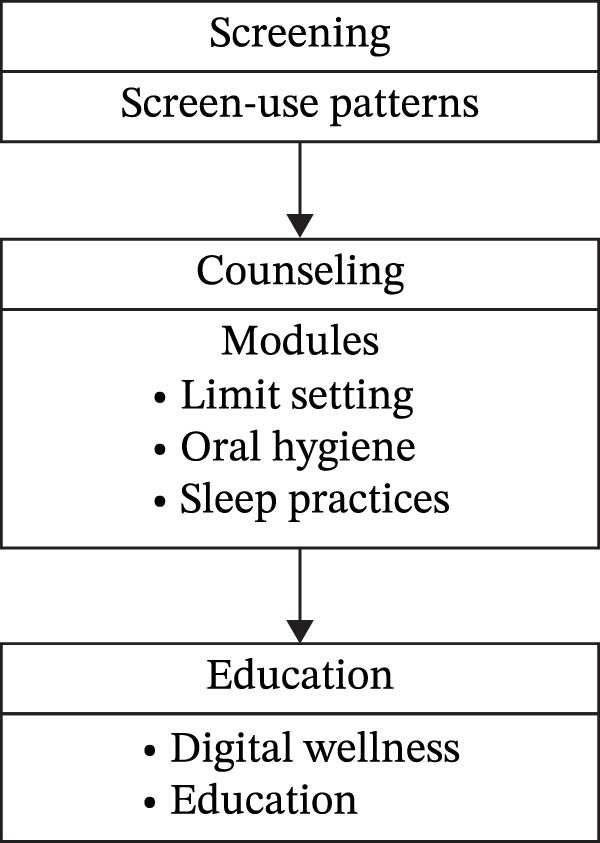
Framework for integrating digital‐behavior counseling in preventive dentistry.

Dentists and pediatricians should integrate digital‐media counseling into standard preventive oral health appointments [14, 16, 19] (Figure 7).

Parental education programs can stress the World Health Organization’s [7] advice to limit children’s recreational screen time to no more than 2 h a day.

Digital wellness campaigns in schools and workplaces can help reduce late‐night device use and the resulting decline in oral hygiene practices [20, 29, 33].

Integrating behavioral economics with oral health promotion could enhance compliance; gamified “digital detox challenges” or mobile reminders to brush after screen time can translate these insights into everyday practice.

### 4.6. Future Directions

Subsequent research should pursue longitudinal and interventional methodologies, incorporating objective screen‐tracking instruments like smartphone analytics or wearable eye‐tracking devices to precisely quantify exposure [28, 33]. Additional investigation into the modulation of the salivary microbiome resulting from light exposure and stress response is necessary [21, 22].

Interdisciplinary frameworks integrating digital psychiatry, chronobiology, and preventive dentistry may provide novel solutions to health challenges in the digital era [29, 32]. Future research should focus on large‐scale community‐based randomized controlled trials that look at how reducing screen time affects oral health. These trials should be done to find out what causes these changes and to make policy‐oriented guidelines.

## 5. Conclusions

This systematic review and meta‐analysis demonstrated that screen exposure was associated with poorer oral‐health outcomes across all age groups, including children, adolescents, and adults [8–15].

Screen exposure is associated with increased odds of dental caries, poorer oral hygiene behaviors, and modest increases in DMFT scores across age groups. These effects were consistent across continents and device types, highlighting a universal behavioral pattern. Mechanistically, dietary mediation, delayed sleep cycles, and reduced salivary secretion may explain the observed associations between digital engagement and oral disease risk [5, 6, 21, 33]. However, given that most included studies were cross‐sectional, these findings should be interpreted as associative rather than causal. Longitudinal studies employing objective measures of screen exposure are required to clarify temporal relationships and potential mechanisms.

### 5.1. Public Health Implications

Although screen exposure was significantly associated with higher odds of dental caries, the cross‐sectional predominance of included studies precludes causal inference. Schools, parents, and healthcare professionals should work collaboratively to include information about digital use in oral health education. Clinicians may consider discussing balanced digital habits as part of broader lifestyle counseling, particularly when addressing dietary and hygiene behaviors.

### 5.2. Future Directions

Longitudinal and interventional studies employing objective screen‐tracking instruments and salivary biomarkers are essential to establish causality and ascertain the reversibility of risk. Integrating digital behavior modification frameworks into preventive dentistry may represent a novel approach in community oral health policy.

## Funding

The authors received no specific funding for this work.

## Conflicts of Interest

The authors declare no conflicts of interest.

## Data Availability

The data that support the findings of this study are available from the corresponding author upon reasonable request.
